# Dataset of internal temperature and gas pressure in cylindrical lithium-ion cells during thermal runaway

**DOI:** 10.1016/j.dib.2025.112190

**Published:** 2025-10-15

**Authors:** Begum Gulsoy, Calum Briggs, Quang Ngo, Mona, Faraji Niri, James Marco

**Affiliations:** a WMG, University of Warwick, Coventry, CV4 7AL, UK; b The Faraday Institution, UK

**Keywords:** Thermal runaway, Internal temperature, Internal gas pressure, Advanced safety characterisation, Cell safety

## Abstract

This experimental dataset relates to the research “*Real-time simultaneous monitoring of internal temperature and gas pressure in cylindrical cells during thermal runaway*” [1]. This study introduces, for the first time, a novel methodology that enables simultaneous real-time monitoring of internal temperature and gas pressure in 21,700 format cylindrical cells. The datasets are from three thermal runaway tests conducted on three instrumented cells, each equipped with an embedded thermocouple and gas pressure adaptor to allow access to internal gasses. Measurements collected during the experiments include cell voltage, internal gas pressure, surface temperatures measured 10 mm from both the negative and positive terminals, surface and internal temperatures at the axial midpoint of the cell, and vent temperatures recorded at 5 mm and 10 mm from the vent cap on the positive terminal. In all experiments, the cells are heated externally using a heating pad until thermal runaway occurs. The evolution of the cell failure has been investigated by monitoring the parameters above and is reported in [1]. This dataset provides insights into the development of thermal runaway events by characterising three main stages: pre-vent, soft-vent and flame generation/explosion. This data serves as a valuable resource for advanced battery failure diagnostics and facilities the development of physical and data-driven models aimed at predicting early detection or developing new mitigation strategies. Moreover, the availability of previously unmeasured battery parameters improves our understanding of battery safety and supports the optimising the design of vent systems for future batteries.

Specifications Table

**Table:** 

Subject	Engineering & Materials science
Specific subject area	Safety characterisation of cylindrical lithium-ion batteries by monitoring internal temperature and gas pressure simultaneously during a thermal runaway event.
Type of data	Processed Table (.mat format)
Data collection	Measurements collected during three thermal runaway tests include cell voltage, internal gas pressure, surface temperatures measured 10 mm from both the negative and positive terminals, as well as surface and internal temperatures at the axial midpoint of the cell. Vent temperatures are recorded at 5 mm and 10 mm from the vent cap on the positive terminal. These parameters were monitored throughout the three key stages of thermal runaway: pre-vent, soft-vent, and flame generation/explosion. All parameters recorded simultaneously using a National Instruments data acquisition system (cDAQ-9178). Voltage-related measurements were logged using an NI 9213 module at a sampling rate of 1 kHz, while temperature-related measurements were recorded using an NI 9220 module at 10 Hz.
Data source location	Institution: Warwick Manufacturing Group (WMG), Energy Innovation Centre, University of Warwick City: Coventry Country: United Kingdom GPS coordinates for collected samples/data: 52.38363378953185, −1.5615186436655097.
Data accessibility	Repository name: Mendeley Data Data identification number: 10.17632/rgfhdhcd9k.2 Direct URL to data: https://data.mendeley.com/datasets/rgfhdhcd9k/1
Related research article	Gulsoy, B., Chen, H., Briggs, C., Vincent, T.A., Sansom, J.E. and Marco, J., 2024. Real-time simultaneous monitoring of internal temperature and gas pressure in cylindrical cells during thermal runaway. Journal of Power Sources, 617, p.235147. https://doi.org/10.1016/j.jpowsour.2024.235147 .

## 1 Value of the Data

• This dataset provides unique, high-resolution measurements of internal cell temperature and gas pressure during thermal runaway, offering rare insights into the failure dynamics of lithium-ion batteries.
• The data captures critical stages of cell failure including pre-vent, soft-vent, and flame/explosion events, enabling a deeper understanding of gas accumulation and heat generation mechanisms.
• The findings support the design of predictive models and diagnostic tools for thermal runaway, which are essential for battery management system (BMS) development.
• Battery manufacturers can leverage this information to enhance cell design, optimize venting systems, and select safer electrode materials, contributing to the next generation of safer, high-performance energy storage systems.

## 2 Background

Lithium-ion batteries are widely used for energy storage and conversion in applications such as consumer electronics, electric vehicles, and emerging aerospace applications. As battery technology advances, new cathode and anode materials are being explored, including nickel- or manganese-rich cathodes and silicon-carbon composite anodes. While these materials offer improved performance, they also introduce increased thermal instability, which raises the risk of safety issues like thermal runaway. This has led to a growing need to better understand the thermal runaway mechanism to develop early detection systems and improve overall battery safety [[1], [2], [3]]. A novel method has been developed to characterise thermal runaway events by simultaneously monitoring internal cell temperature and gas pressure during key failure stages: pre-vent, soft-vent, and flame generation or explosion. The resulting dataset provides important insights into gas accumulation and temperature rise during cell failure. These findings support the development of diagnostic tools and predictive models, and improved battery design and venting systems [1].

## 3 Data Description

**Fig. 1:**
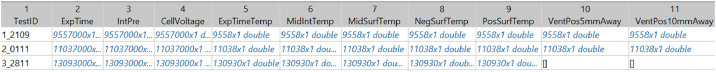
Structured dataset in .mat format.

The experimental dataset comprises three thermal runaway tests conducted on instrumented Sony VTC6A cells. The dataset was processed and saved in MATLAB data (*.mat*) format. Fig. 1 shows the structured dataset.

The dataset includes measurements of cell voltage, internal gas pressure, surface temperatures measured 10 mm from both the negative and positive terminals, surface and internal temperatures at the axial midpoint of the cell, and vent temperatures recorded at 5 mm and 10 mm from the vent cap on the positive terminal, as detailed in Table 1. All parameters except *IntPre* collected directly from the data logger. *IntPre* was measured as a voltage signal and converted to pressure in bar using a transfer function derived from calibration of the pressure sensor.

**Table 1:** Parameters measured during thermal runaway testing.

Column Number	Parameters	Unit	Details
1	*TestID*	−	Test ID assigned to each test cell
2	*ExpTime*	seconds	Duration of test measured in seconds, starting from the beginning of experiment. This time variable is used for *IntPre* and *CellVoltage*.
3	*IntPre*	bar	Internal gas pressure measured during testing
4	*CellVoltage*	volt	Cell voltage measured during testing
5	*ExpTimeTemp*	seconds	Duration of test measured in seconds for temperature-rated measurements, starting from the beginning of the experiment
6	*MidIntTemp*	°C	Internal cell temperature at the axial midpoint of the cell
7	*MidSurfTemp*	°C	Surface cell temperature at the axial midpoint of the cell
8	*NegSurfTemp*	°C	Surface cell temperature measured at a point 10 mm away from the negative terminal
9	*PosSurfTemp*	°C	Surface cell temperature measured at a point 10 mm away from the positive terminal
10	*VentPos5mmAway*	°C	Vent temperature measured 5 mm away from the vent cap at the positive terminal
11	*VentPos10mmAway*	°C	Vent temperature measured 10 mm away from the vent cap at the positive terminal

## 4 Experimental Design, Materials and Methods

### 4.1 Cell information

The Sony VTC6A is a cylindrical lithium-ion cell in the 21,700-format, featuring a rated capacity of 4 Ah. It has a nominal voltage of 3.6 V and an average mass of approximately 67.37 ± 0.5 g The cell has a charge cut-off voltage of 4.25 V and a discharge cut-off voltage of 2.5 V. Its operating temperature ranges are 0 °C to +60 °C for charging and −20 °C to +60 °C for discharging. The cell utilizes a Lithium Nickel Cobalt Aluminium Oxides (NCA) cathode, a Graphite–Silicon Oxide (SiOx) composite anode. The electrolyte consists of lithium hexafluorophosphate (LiPF₆) salt dissolved in a mixture of dimethyl carbonate (DMC) and ethylene carbonate (EC) organic solvents. As part of their safety mechanism, the cells are equipped with a negative-end vent in addition to the conventional top venting disk [1].

### 4.2 Cell preparation

All cells were instrumented with embedded thermocouples and pressure transducers using the methodology described in our previous publications [2,3]. Prior to testing, all cells were charged to their upper voltage limit to achieve 100 % SOC using a CC—CV charging protocol. During the CC phase, a current of 1C was applied, followed by a CV phase at a voltage of 4.25 V, that continued until the current reduced to C/20. This ensured consistent SOC conditioning across all test samples.

### 4.3 Thermal runaway tests

**Fig. 2:**
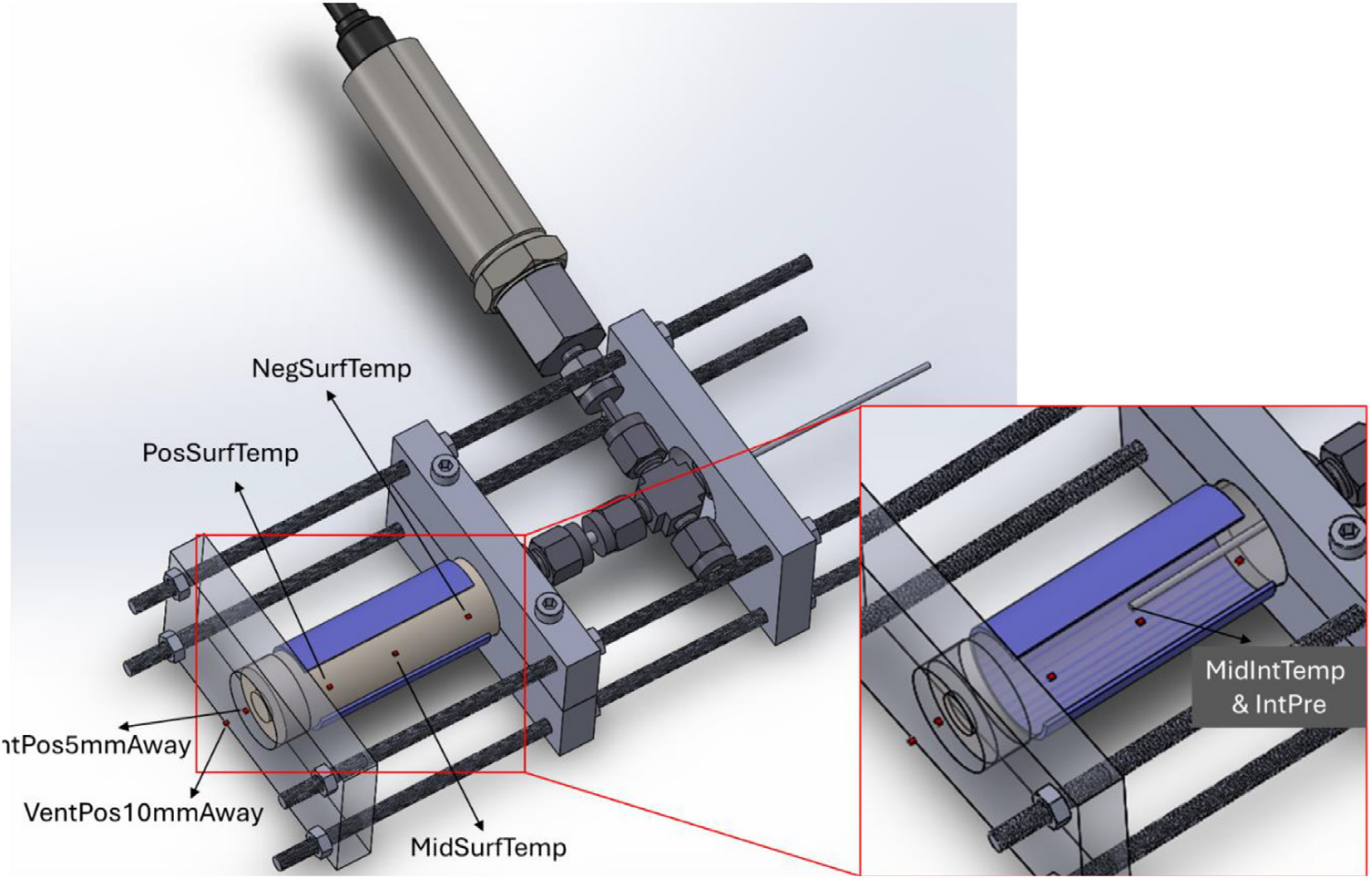
Schematic of the instrumented cell mounted on a bespoke test fixture, showing the locations of temperature and internal gas measurements.

Thermal runaway tests were initiated by an external heating method. A flexible heating pad (50 mm x 50 mm) was placed on the cell surface, and a constant heating power of 40 W was applied via an external power supply until the onset of thermal runaway. All instrumented cells were secured with a bespoke test fixture (Fig. 2). Experiments were conducted within a dedicated abuse testing facility, where the ambient temperature ranged between 15 °C and 20 °C. Further details of the test setup are provided in [1] and are therefore not repeated here.

All test parameters were recorded simultaneously using a National Instruments data acquisition system (cDAQ-9178). Voltage-related measurements, including *ExpTime, IntPre*, and *CellVoltage*, were logged using an NI 9213 module at a sample rate of 1 kHz, while temperature-related measurements (*ExpTimeTemp, MidIntTemp, MidSurfTemp, NegSurfTemp, PosSurfTemp, VentPos5mmAway*, and *VentPos10mmAway*) were recorded using an NI 9220 module at a sampling rate of 10 Hz. Fig. 2 shows the locations of the measurements corresponding to the parameters discussed.

## Limitations

Not applicable.

## Ethics Statement

The proposed data does not involve any human subjects, animal experiments, or data collected from social media platforms. The authors confirm that this work meets the ethical requirements of the journal.

## Credit Author Statement

**B. Gulsoy:** Conceptualization, Methodology, Formal analysis, Investigation, Writing - Original Draft. **C. Briggs:** Methodology, Investigation. **Q. Ngo:** Data Processing, Formal analysis. **M.F. Niri:** Data Processing, Writing - Review & Editing. **J. Marco:** Funding acquisition, Supervision, Resources, Writing - Review & Editing.

## Data Availability

Mendeley DataDataset of internal temperature and gas pressure in cylindrical lithium-ion cells during thermal runaway (Original data).
